# Autologous distal femoral condyle graft for patellar reconstruction during TKA in posttraumatic ankylosis: a case report

**DOI:** 10.3389/fsurg.2026.1791207

**Published:** 2026-06-01

**Authors:** Chengzhi Dai, Liping Pan, Yongping Cao, Qiwei Wang, Shihao Hong

**Affiliations:** Department of Orthopaedics, Peking University First Hospital, Beijing, China

**Keywords:** ankylosis, case report, patellar reconstruction, patellectomy, total knee arthroplasty

## Abstract

**Introduction:**

Post-traumatic knee ankylosis after patellectomy is uncommon and technically challenging to reconstruct. Loss of the patella shortens the quadriceps moment arm, thereby impairing extensor strength. In active patients, total knee arthroplasty (TKA) alone may leave residual anterior symptoms unresolved.

**Case presentation:**

A 48-year-old woman presented with severe stiffness and pain after prior patellectomy, with a range of motion (ROM) of 0°–15°. Imaging showed advanced osteoarthritis without obvious collateral deficiency. She underwent TKA combined with implantation of an autologous distal femoral condyle graft as a patellar substitute, with additional suture-anchor reinforcement of the patellar tendon insertion. At 10 months postoperatively, ROM was 0°–120°, the 2011 Knee Society Score (KSS) improved (pain: 40 → 50; function: 13 → 69), and no clinical instability was observed.

**Discussion:**

Patellar absence reduces extensor efficiency and anterior stability; reconstruction aims to restore extensor continuity and patellofemoral tracking. Ankylosis requires stepwise soft-tissue release and careful joint-line management. Autologous bone graft offers potential for biological incorporation but carries risks of resorption or nonunion. Implant constraint should be minimized while maintaining adequate stability.

**Conclusion:**

In this patient with post-patellectomy knee ankylosis, TKA combined with autologous patellar reconstruction was feasible and was associated with acceptable short-term clinical stability and functional improvement in the context of combined ankylosis conversion TKA. Long-term durability and any potential advantage over TKA alone require evaluation in larger studies with extended follow-up.

## 1 Introduction

Post-traumatic knee ankylosis after severe injury is uncommon but can cause marked functional limitation. Although TKA can restore joint motion, stiff or ankylosed knees typically require extensive soft-tissue releases and meticulous flexion–extension gap balancing. Accordingly, postoperative instability and complication rates are higher than those after primary TKA in standard cases (1, 2). When prior patellectomy has been performed, reconstruction becomes even more challenging. Loss of this mechanical fulcrum shortens the effective moment arm of the extensor mechanism, adversely affecting active extension and functional stability.

Available evidence suggests that patients with prior patellectomy can achieve clinically meaningful functional improvement after TKA; however, outcomes and revision risk may vary by implant design and patient-specific factors (3). Despite advances in prosthetic design and surgical technique, anterior knee pain and patellofemoral-related problems remain leading causes of dissatisfaction after TKA. These issues are closely related to preoperative limb alignment, soft-tissue balance, and the mechanical properties of the extensor mechanism (4, 5).

In this context, patellar reconstruction during TKA should be considered a selective adjunct rather than a routine procedure. It may be appropriate in patellectomized knees in which TKA alone cannot adequately restore extensor efficiency or anterior stability, or cannot meet higher functional demands. Autologous bone graft may provide biological advantages, including osteogenic and osteoconductive potential, while avoiding risks associated with allograft. Autologous bone graft may provide biological advantages, but the clinical behavior of such a construct depends on graft size, soft-tissue containment, host biology, and postoperative follow-up (6, 7).

This report describes a complex case of post-patellectomy traumatic knee ankylosis treated with TKA combined with an autologous distal femoral condyle graft for patellar reconstruction. In accordance with the SCARE 2025 reporting guidelines (8), we discuss the surgical rationale—including considerations regarding tibial posterior slope—fixation strategy, and early clinical outcomes, together with an analysis of the inherent limitations of this approach.

## 2 Presentation of case

### 2.1 Clinical history

A 48-year-old woman presented with a 20-year history of progressive left knee stiffness and anterior knee pain, which had worsened over the preceding 6 months with activity.

Twenty years earlier, the patient sustained an open left knee injury and underwent plate-and-screw internal fixation with concurrent patellectomy at an outside institution. Details of the injury pattern and operative procedure were unavailable due to the long interval. After surgery, the knee was immobilized in extension for a prolonged period, followed by progressive stiffness and a gradual decline in active ROM. Internal fixation hardware was removed 1 year postoperatively. Approximately 2 years after the initial injury, arthrolysis was performed, temporarily restoring ROM to approximately 0°–90°; however, stiffness recurred and progressively worsened over subsequent years, declining to 0°–15°. Over the following two decades, the patient developed severe functional limitation, including a stiff-legged gait and substantial difficulty climbing stairs, walking long distances, and rising from a seated position. Six months before presentation, anterior knee pain intensified without an obvious precipitating cause, was exacerbated by weight-bearing, and was only partially relieved by rest. Conservative management—including oral analgesics and physiotherapy—provided limited relief.

Physical examination showed varus alignment of the left lower extremity with a stiff gait. Multiple well-healed surgical scars were present over the anterior knee, and no patella was palpable. Medial joint-line tenderness was pronounced, without erythema or swelling, and no significant effusion was detected. Active and passive ROM of the left knee was 0°–15° without an extension lag. The contralateral knee had full ROM without pain. Distal muscle strength, sensation, and peripheral pulses were normal bilaterally.

**Figure 1:**
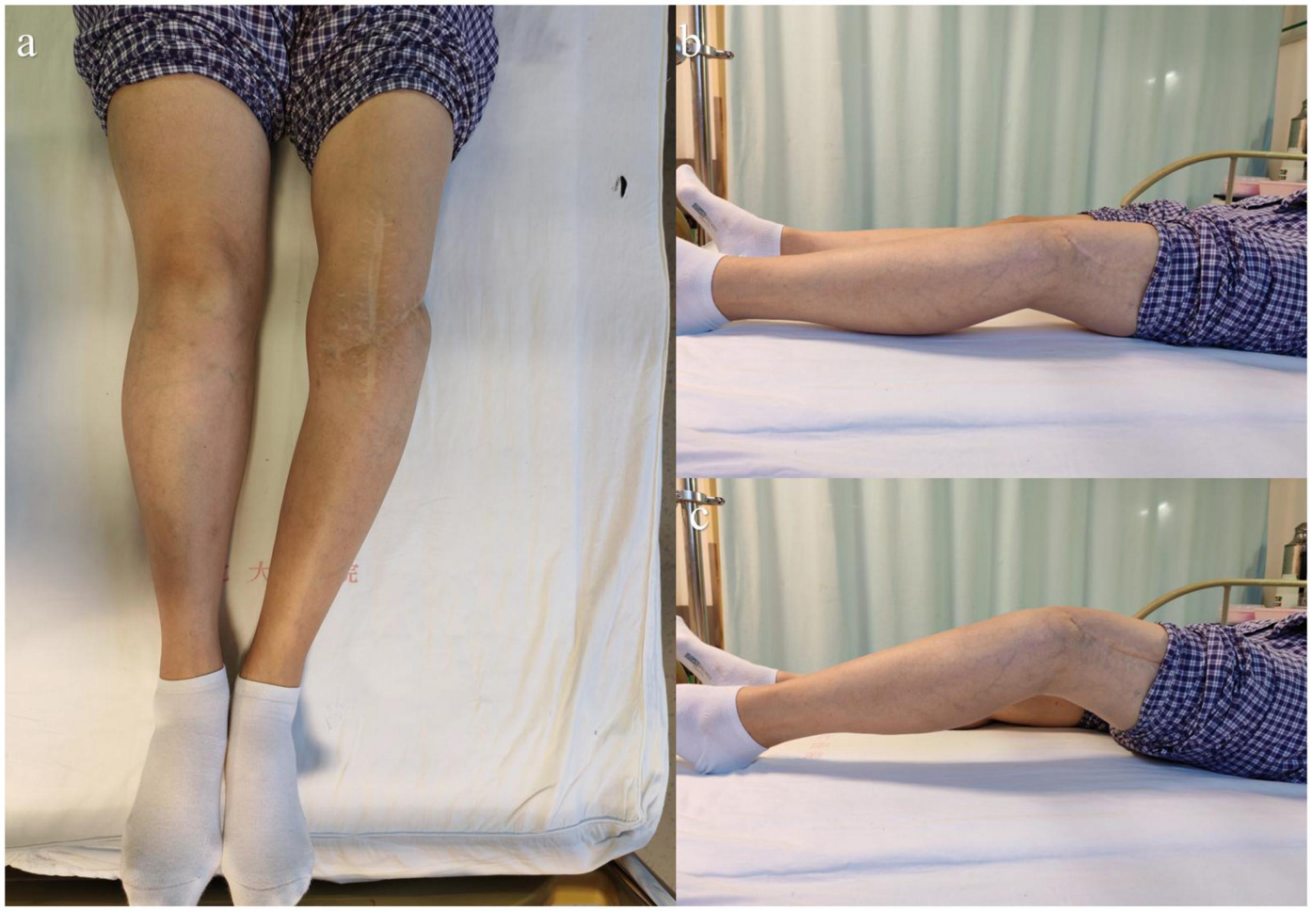
Preoperative clinical photographs. **(a)** Supine anterior view showing varus alignment of the left knee, a midline scar from prior patellectomy, and marked quadriceps wasting, most pronounced in the vastus medialis. **(b)** Lateral view at attempted full extension: no extension lag; prepatellar skin depression with reduced quadriceps tone. **(c)** Maximal active flexion limited to ∼15°, below the functional range and consistent with severe stiffness.

**Figure 2:**
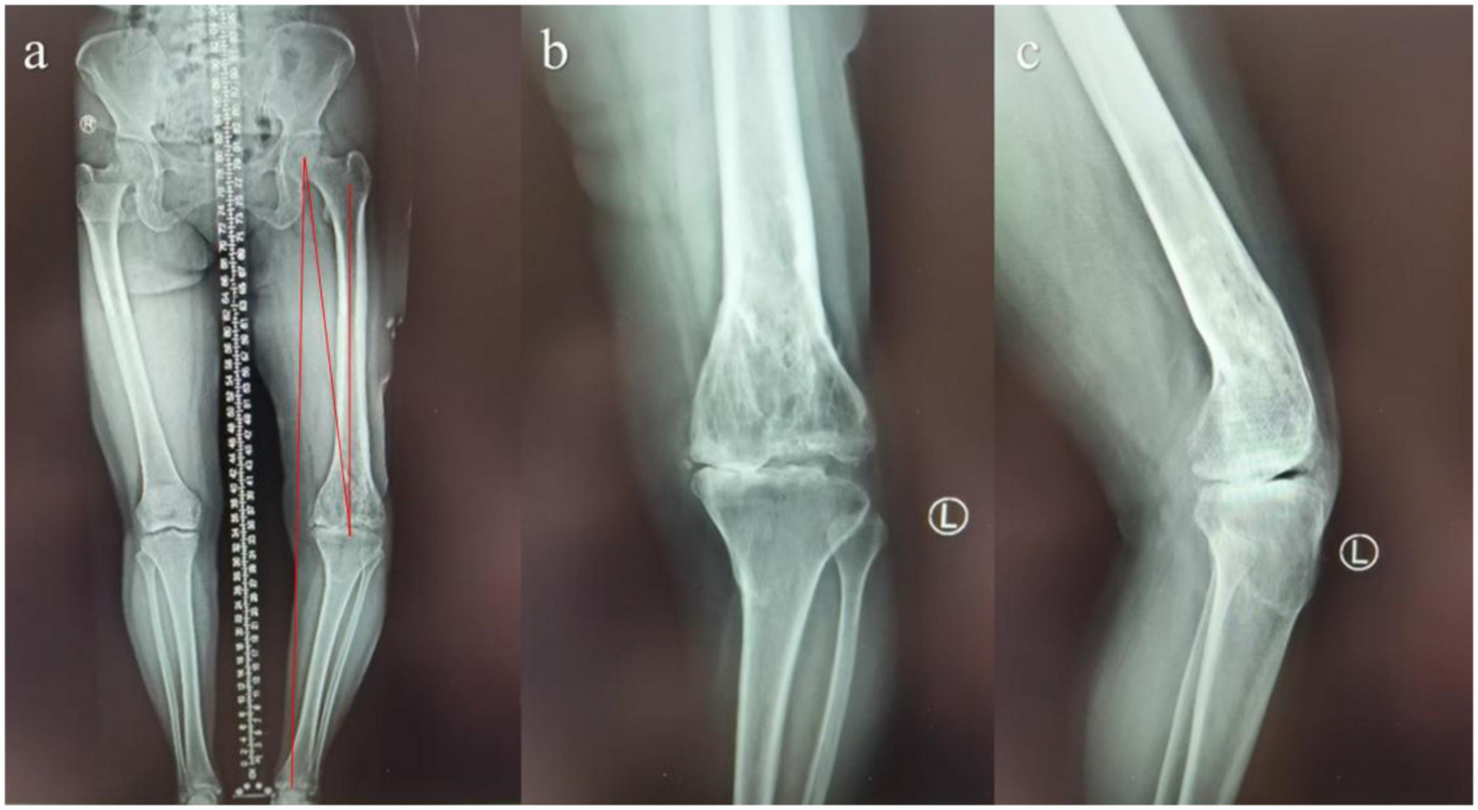
Preoperative imaging. (**a**) Preoperative standing full-length lower limb radiograph showing a 1° varus alignment based on the hip–knee–ankle angle. The femoral mechanical axis demonstrated 7° valgus, with a femoral mechanical–anatomical axis difference of approximately 7°. (**b**) Anteroposterior radiograph of the left knee showing severe post-traumatic osteoarthritis and absence of the patella. (**c**) Lateral radiograph of the left knee showing post-traumatic degenerative change and patellar absence.

Plain radiographs demonstrated severe post-traumatic osteoarthritis of the left knee with complete absence of the patella and varus deformity. Standing full-length lower limb radiographs confirmed mechanical axis deviation (measurements detailed below). Given the prolonged severe stiffness, marked functional impairment, progressive pain, and failure of conservative treatment, TKA with concurrent patellar reconstruction was planned. Preoperative clinical photographs and imaging findings are shown in Figures 1, 2.

### 2.2 Total knee arthroplasty

Under general anesthesia with tourniquet control, TKA with concurrent patellar reconstruction was performed through a standard anterior midline incision and medial parapatellar approach. Severe ankylosis with dense adhesions was encountered. To improve exposure and flexion, proximal mobilization of the quadriceps-extensor mechanism and subperiosteal release of the medial soft tissues from the proximal tibia were performed. The patellar tendon insertion was preserved and protected with a temporary Kirschner wire.

A tibial resection of approximately 10 mm with 5° posterior slope was performed first, followed by distal femoral resection of approximately 10 mm at 6° valgus using an intramedullary guide. Trial reduction with a size 4 posterior-stabilized femoral component, size D tibial baseplate, and 8 mm polyethylene insert demonstrated satisfactory gap balance, stable motion, and no significant mediolateral instability. The final components were then cemented in place after irrigation.

### 2.3 Patellar reconstruction

An autologous corticocancellous bone block harvested from the distal femoral condyle resection surface was used as a patellar substitute. The graft was trimmed to an appropriate contour and size (approximately 3 cm × 4 cm with a thickness of about 1 cm). A longitudinal split was then made in the quadriceps tendon approximately 15 mm distal to the superior margin of the femoral component, creating a pouch for graft insertion. The prepared bone block was inserted into this pouch and secured by suture fixation.

**Figure 3:**
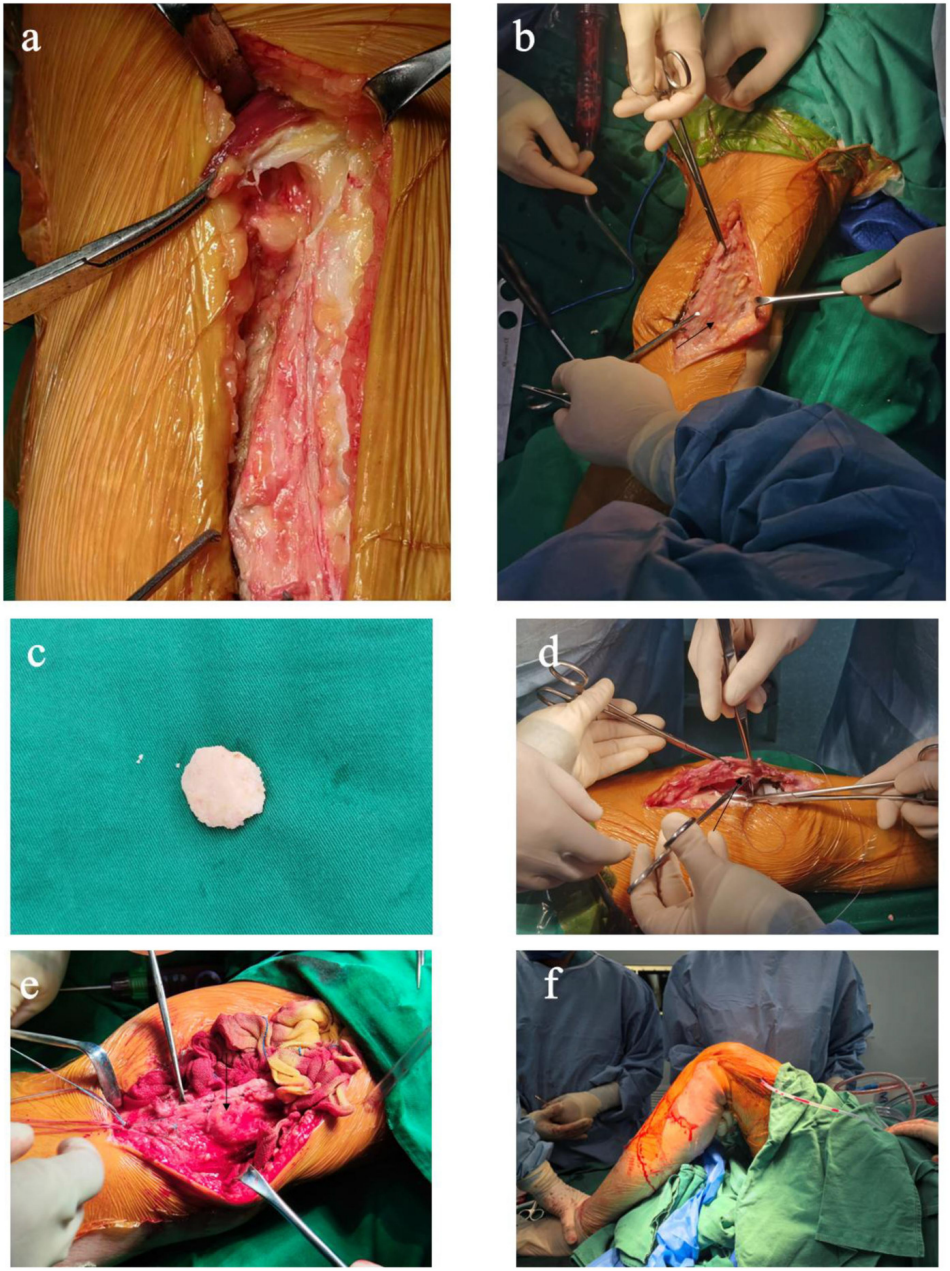
Intraoperative photographs. **(a)** Medial parapatellar exposure demonstrating dense intra-articular adhesions in a long-standing ankylosed knee. **(b)** Intraoperative confirmation of patellar absence with preservation of the patellar tendon insertion (arrow). **(c)** Autologous bone graft harvested from the distal femoral condyle and shaped into a dome configuration to approximate native patellar contour. **(d)** Creation of a central intratendinous pocket within the quadriceps tendon for graft implantation. **(e)** Morphological appearance of the reconstructed patella after implantation and fixation. **(f)** Functional assessment of the reconstructed extensor mechanism demonstrating central patellar tracking during knee flexion; the no-thumb test was negative.

To reinforce the extensor mechanism, two medial-row suture anchors and one lateral-row suture anchor were used to augment fixation of the patellar tendon insertion. These anchors were intended to strengthen the distal attachment of the extensor mechanism rather than to directly fix the graft itself. Patellar tracking was then assessed and found to be satisfactory, with a negative no-thumb test. Cocktail analgesia was injected around the joint capsule and pes anserinus region. After thorough irrigation, the wound was closed in layers. Key intraoperative steps of the arthroplasty and patellar reconstruction are shown in Figure 3.

### 2.4 Postoperative course and follow-up

Early postoperative rehabilitation focused on swelling management, low-molecular-weight heparin anticoagulation, and ankle-pumping exercises. Assisted standing and ambulation were initiated on postoperative days 1–2, and the drain was removed at 48 h. From postoperative day 3 through week 2, progressive rehabilitation included straight-leg raises, isometric quadriceps contractions, and seated flexion–extension exercises. By postoperative week 2, the patient was able to ambulate independently on level ground; by week 4, knee flexion ROM had reached approximately 90°.

**Figure 4:**
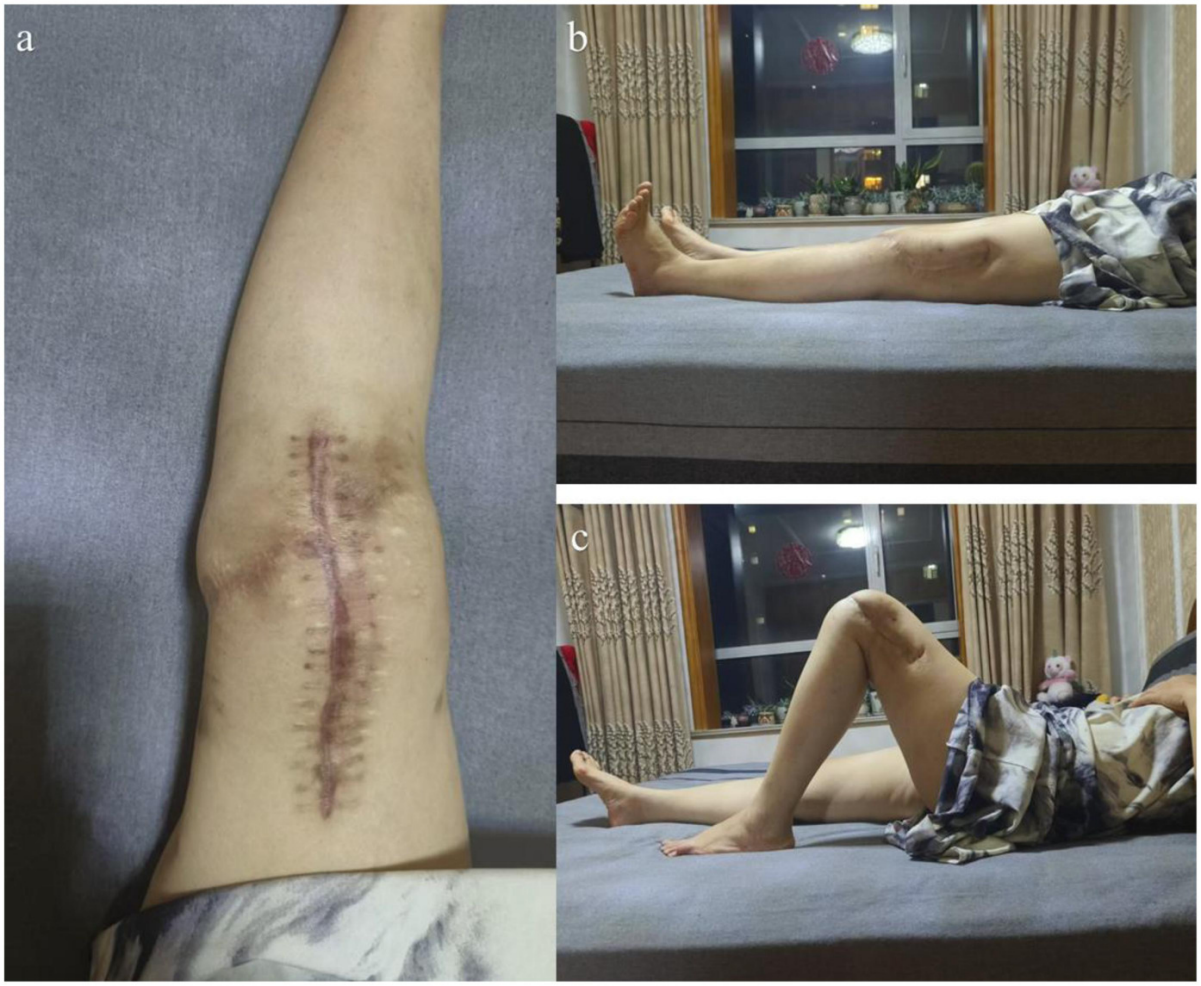
Clinical photographs at 10-month follow-up. **(a)** Anterior view: healed midline scar; neutral coronal alignment; no visible quadriceps wasting. **(b)** Lateral view in full extension (0°): no extension lag; no prepatellar skin depression. **(c)** Lateral view in maximal flexion (∼120°).

**Figure 5:**
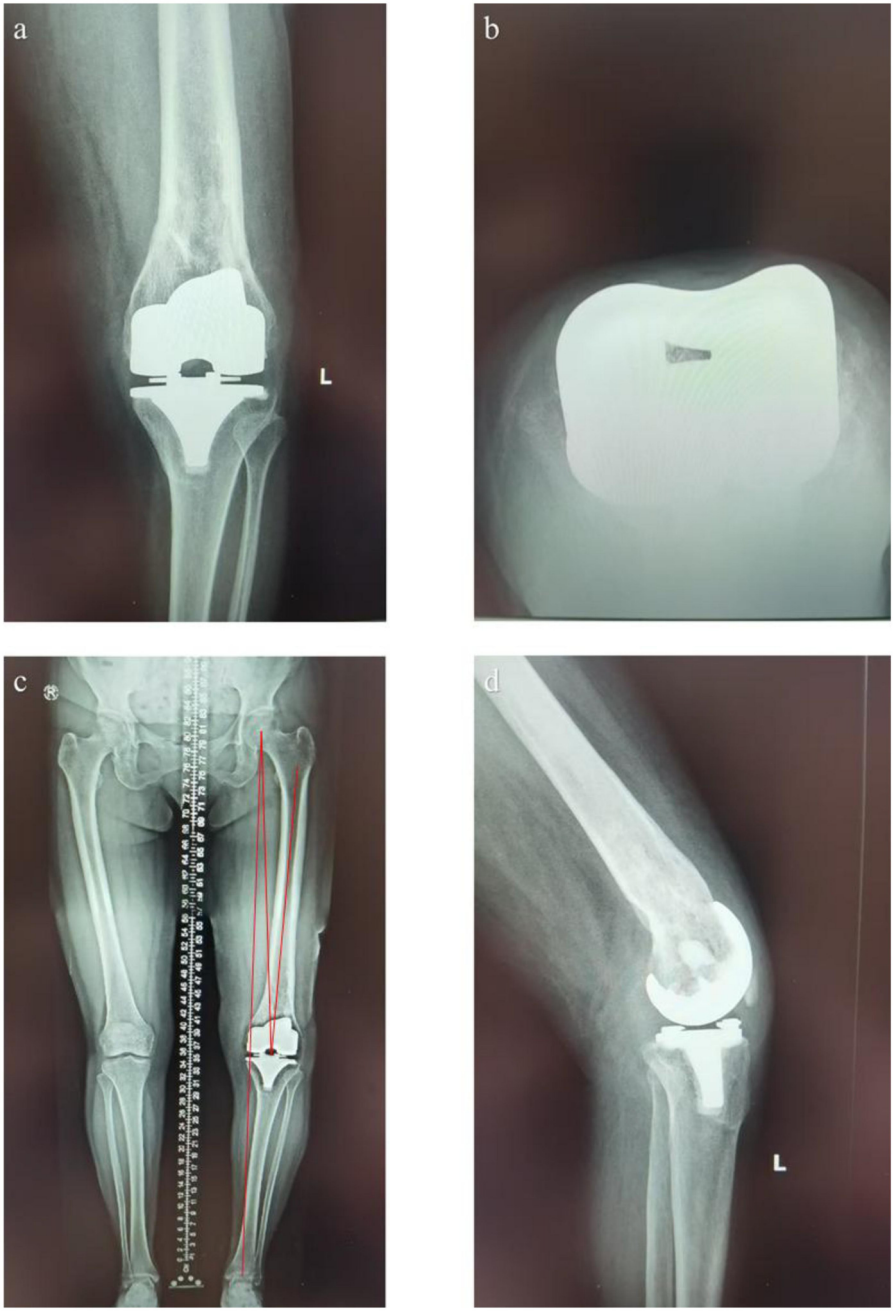
Postoperative radiographs at 10-month follow-up. (**a**) Anteroposterior radiograph of the left knee after total knee arthroplasty and patellar reconstruction. (**b**) Merchant/sunrise view showing the reconstructed anterior patellar substitute and prosthetic components. (**c**) Postoperative full-length standing radiograph showing a residual 2° varus alignment of the limb based on the hip–knee–ankle angle, with the femoral mechanical axis oriented at 2° valgus and an estimated mechanical–anatomical axis difference of approximately 7°. (**d**) Lateral radiograph of the left knee showing the prosthetic components and reconstructed anterior patellar substitute.

At the 10-month follow-up, the patient reported no anterior knee pain and no instability. Physical examination showed ROM of 0°–120°, independent stair climbing, and no flexion contracture or extension lag. The patient reported satisfaction with the outcome and markedly improved confidence in daily activities compared with the preoperative state. Using the 2011 revised Knee Society Score (KSS), the pain score improved from 40 to 50 points, and the function score improved from 13 to 69 points; the stability and ROM subscores also improved relative to preoperative values. No perioperative or early postoperative complications were identified during follow-up, including wound-related complications, infection, thromboembolic events, or the need for reoperation. Clinical and radiographic findings at the 10-month follow-up are shown in Figures 4, 5.

Preoperatively, gait was antalgic with a stiff-limb strategy on the involved side. The patient demonstrated reduced ipsilateral step length and stance time and maintained a consistently downward gaze during ambulation. In the sagittal plane, the knee posture during stance was near-neutral or mildly hyperextended, consistent with a stiff-knee pattern. Hip hiking (ipsilateral pelvic elevation during swing) and forward trunk inclination during stance were visually apparent.

At 10 months postoperatively, overall gait quality improved compared with baseline. During stance, the knee demonstrated a more flexed posture during loading response than preoperatively. During swing, the knee flexion arc appeared increased. Hip hiking was reduced but remained present to a mild degree. Forward trunk inclination was reduced, with a more upright trunk posture during ambulation, although mild anterior trunk lean persisted. Gaze direction remained downward.

These findings were derived from observational assessment and were not instrumented or quantified. The observational gait features before and after surgery are summarized in Table 1.

**Table 1:** Summary of observational gait features.

Kinematic parameter	Preop status	10-Month postop	Change
Stance-phase knee posture	Near-neutral or mild hyperextension	More flexed posture during loading response	Improved
Swing-phase knee flexion arc	Visually reduced	Visually increased	Improved
Hip hiking (ipsilateral pelvic elevation during swing)	Present	Reduced, mild residual	Partially improved
Trunk anterior flexion during stance	Forward trunk inclination	Reduced	Improved

## 3 Discussion

Management of post-patellectomy knee ankylosis requires addressing two interrelated objectives: first, resolving ankylosis, restoring the joint line, and recovering range of motion (ROM) through total knee arthroplasty (TKA); and second, compensating, at least partially, for the loss of extensor mechanism efficiency caused by patellar absence. In long-standing ankylosis or severe stiffness, postoperative ROM improvement primarily results from ankylosis conversion, stepwise soft-tissue release, restoration of joint-line and limb-alignment relationships, and postoperative rehabilitation, and should not be attributed to any single adjunctive procedure (2). Accordingly, the substantial ROM gain in this case most likely reflects the combined effects of ankylosis conversion and standardized rehabilitation; the patellar reconstruction component should therefore be interpreted mainly as part of an extensor mechanism reconstruction strategy, providing anterior structural support and continuity rather than serving as the principal determinant of ROM recovery.

Biomechanically, patellar absence reduces extensor mechanism efficiency, impairs knee function, and may contribute to weakness or a subjective sense of instability after TKA (3). In addition, anterior knee pain and patellofemoral-related problems remain important contributors to dissatisfaction after surgery, and abnormal patellar tracking or instability may further worsen clinical outcomes (4, 5). Patellofemoral mechanical mismatch can produce anterior knee pain: any factor causing eccentric loading of the patella, or of a reconstructed patellar substitute, within the trochlear groove—including femoral or tibial component malrotation, trochlear geometry mismatch, soft-tissue imbalance, joint-line alteration, or an abnormal Q-angle and limb alignment—may present as lateral tilt, subluxation, or dislocation, and may clinically manifest as anterior knee pain, crepitus, weakness, or instability (5). In the present case, the patient showed marked postoperative ROM improvement and a modest improvement in pain score, which may reflect relief of anterior soft-tissue symptoms, restoration of alignment, and improved overall knee mechanics after reconstruction.

Implant selection in post-patellectomy ankylosed knees requires balancing stability against the risks associated with higher constraint. A systematic review of 9 studies including 209 knees (mean interval from patellectomy to TKA: 16.1 years) reported significant functional improvement with both cruciate-retaining (CR) and posterior-stabilized (PS) implants. The relative improvement in Knee Society Score (KSS) was greater with CR than with PS implants (+108% vs. +98%, *P* ≤ 0.001), but the revision rate was higher in the CR group than in the PS group (18.6% vs. 2.6%, *P* = 0.002) (3). In the present case, given the extensive soft-tissue release required for ankylosis conversion, posterior cruciate ligament (PCL) function and tension were considered insufficient; therefore, a PS implant was selected to substitute for the PCL and provide more reproducible flexion kinematics. Intraoperative gap balance was satisfactory, collateral ligament function was acceptable, and no notable coronal-plane instability was observed, so higher-constraint constructs such as a condylar-constrained knee (CCK) or hinged prosthesis were not required. In cases with collateral ligament insufficiency, severe bone defects, or difficulty achieving gap balance, the level of constraint should be escalated according to stability requirements.

Tibial posterior slope (TPS) influences sagittal-plane kinematics and flexion-gap behavior. In general, increasing TPS tends to loosen the flexion gap, facilitating greater flexion and potentially reducing the quadriceps force and patellofemoral contact force required during knee extension; however, it also increases anterior tibial translation relative to the femur, thereby altering stability and contact-stress distribution (9). In PS implants, excessive TPS may increase anterior tibial shift and cam-post loading, raising the risk of instability or impingement. A musculoskeletal/computer simulation study reported that increasing TPS may improve extensor efficiency and reduce quadriceps force and patellofemoral contact force, but may also increase the risks of anterior tibial component migration and anterior post impingement; the authors recommended avoiding excessive TPS and suggested TPS <5° for greater safety in PS TKA (10). More recent modeling studies support the concept that TPS systematically alters joint mechanics and soft-tissue loading. A subject-specific musculoskeletal modeling study found that TPS changes significantly affect joint contact mechanics and soft-tissue load distribution (11), while a finite element study suggested that TPS and tibial rotational alignment interact to influence intra-articular stress and cam-post loading in PS prostheses (12). Therefore, TPS should be controlled within the prosthesis-recommended range and adjusted according to intraoperative gap and stability assessment, rather than increased simply to maximize flexion. In the present case, TPS was set at 5° because, after ankylosis conversion and extensive soft-tissue release, sufficient flexion-gap margin was needed to facilitate early flexion recovery while avoiding the high-risk zone of excessive slope, anterior tibial shift, increased cam-post loading, and potential instability.

With respect to patellar reconstruction, prior reports have primarily described it as a structural compensatory measure for patellar absence, intended to improve extensor mechanism continuity and optimize the mechanical environment of the anterior knee (13, 14). Distal femoral autograft used as a reconstructed patellar body is among the most frequently reported techniques (13, 14). More recent case reports have also described autologous patellar reconstruction at the time of TKA using either femoral condylar autograft or bone obtained from routine cuts, but the available evidence remains limited to technical reports and isolated cases without comparative controls or instrumented functional assessment (15). Therefore, this approach is best regarded as a selective option for specific circumstances rather than a broadly generalizable standard procedure (14).

Importantly, the present case should not be interpreted as an anatomic restoration of a native patella. In our procedure, the autologous corticocancellous bone block served as a patellar substitute: after shaping, it was inserted into a longitudinal pouch created within the quadriceps tendon approximately 15 mm distal to the superior margin of the femoral component and secured with sutures. The distal extensor mechanism was then reinforced using two medial-row and one lateral-row suture anchors to augment fixation at the patellar tendon insertion. Thus, the suture anchors were used to reinforce the patellar tendon insertion rather than to directly fix the graft itself. From a biomechanical perspective, this construct more likely provided localized anterior structural support and improved extensor mechanism continuity than fully recreated native patellofemoral biomechanics. Given the limited graft thickness (approximately 1 cm), this reconstruction should be understood as a partial patellar substitute rather than a true anatomic recreation of the native patella. The potential contribution of this reconstruction to extensor lever-arm restoration or anterior stability cannot be quantified directly in the present case.

The distinction between release plus TKA alone and release plus patellar reconstruction also warrants emphasis. In ankylosed knees, extensive release and arthroplasty alone are already expected to substantially improve ROM, pain, and gait mechanics (2). Therefore, the observed postoperative functional improvement in this patient cannot be confidently attributed to patellar reconstruction alone. Nevertheless, in a knee with previous patellectomy, implantation of a localized anterior bone block together with reinforcement of the patellar tendon insertion may still have value in restoring a more continuous anterior extensor mechanism profile and in reducing soft-tissue instability during knee motion. Intraoperatively, tracking of the reconstructed anterior mechanism was satisfactory and the no-thumb test was negative, suggesting acceptable short-term mechanical behavior; however, this should be interpreted cautiously and not as proof of restored normal patellofemoral mechanics.

The postoperative observational gait improvements noted in this case—including more physiologic knee flexion during loading response, an increased swing-phase knee flexion arc, and reduced hip hiking and trunk lean—are clinically encouraging, but assessment was non-instrumented, and no definitive conclusions regarding extensor efficiency can be drawn. These changes cannot be confidently attributed to patellar reconstruction alone, because major concurrent drivers, including ankylosis conversion, extensive soft-tissue release, restoration of joint mechanics with TKA, pain reduction, and rehabilitation, are all independently expected to improve gait. The best available contemporary clinical support for reconstruction in patellectomized TKA remains low-level evidence, mainly case reports and technical reports describing improved functional performance and walking tolerance after autologous patellar reconstruction performed at the time of TKA, but without instrumented gait quantification or comparative controls (15).

This report has notable limitations. A follow-up of only 10 months is insufficient to assess long-term graft behavior and implant survivorship. No instrumented or quantitative assessments of quadriceps strength, extension torque, stair performance, or gait were performed; therefore, conclusions regarding recovery of extensor function remain inferential. In addition, postoperative three-dimensional computed tomography (CT) or dynamic Merchant views at different flexion angles would have provided stronger evidence regarding graft morphology, tracking, and integration; however, such imaging was not available because it was not covered and was not clinically justified within the routine follow-up pathway. The absence of these assessments is an important limitation. Accordingly, this case should be interpreted as demonstrating technical feasibility and acceptable short-term clinical outcome in a rare and complex setting, rather than definitive restoration of extensor mechanism biomechanics. This strategy may be more suitable for selected patients in whom the local soft-tissue envelope and patellar tendon insertion can be adequately preserved and reinforced.

## 4 Conclusion

In this patient, distal femoral condyle autograft patellar reconstruction combined with simultaneous TKA resulted in notable functional improvement at the 10-month follow-up: knee ROM reached 0°–120°, and the KSS function score improved from 13 to 69 points. Intraoperatively, reconstruction was used to improve extensor mechanism continuity and provide anterior structural support within the overall reconstructive strategy.

The limitations of this report include the single-case design, short follow-up duration, lack of imaging evidence confirming graft incorporation, and the absence of instrumented or quantitative assessments of quadriceps strength, extension torque, or gait. Future prospective studies with larger sample sizes and longer follow-up are needed to validate the durability of this reconstructive strategy and long-term implant survivorship.

## Data Availability

The original contributions presented in the study are included in the article/Supplementary Material, further inquiries can be directed to the corresponding author.
